# Efficacy and safety of ravidasvir plus sofosbuvir in patients with chronic hepatitis C infection without cirrhosis or with compensated cirrhosis (STORM-C-1): interim analysis of a two-stage, open-label, multicentre, single arm, phase 2/3 trial

**DOI:** 10.1016/S2468-1253(21)00031-5

**Published:** 2021-04-16

**Authors:** Isabelle Andrieux-Meyer, Soek-Siam Tan, Sombat Thanprasertsuk, Nicolas Salvadori, Caroline Menétrey, François Simon, Tim R Cressey, Hajjah Rosaida Hj Mohd Said, Muhammad Radzi Abu Hassan, Haniza Omar, Hoi-Poh Tee, Wah Kheong Chan, Suresh Kumar, Satawat Thongsawat, Kanawee Thetket, Anchalee Avihingsanon, Suparat Khemnark, Sabine Yerly, Nicole Ngo-Giang-Huong, Sasikala Siva, Alistair Swanson, Vishal Goyal, Francois Bompart, Bernard Pécoul, Shahnaz Murad

**Affiliations:** a Drugs for Neglected Diseases initiative, Geneva, Switzerland; b Department of Hepatology, Selayang Hospital, Batu Caves, Malaysia; c Department of Disease Control, Ministry of Public Health, Bangkok, Thailand; d Public Health Promotion Research and Training-Institut de Recherche pour le Développement, Faculty of Associated Medical Sciences, Chiang Mai University, Chiang Mai, Thailand; e Department of Molecular & Clinical Pharmacology, University of Liverpool, Liverpool, UK; f Gastroenterology & Hepatology Unit, Ampang Hospital, Ampang Jaya, Malaysia; g Gastroenterology Unit, Medical Department, Hospital Sultanah Bahiyah, Alor Setar, Malaysia; h Gastroenterology Unit, Medical Department, Hospital Tengku Ampuan Afzan, Kuantan, Malaysia; i Gastroenterology and Hepatology Unit, Department of Medicine, Faculty of Medicine, University of Malaya, Kuala Lumpur, Malaysia; j Infectious Disease Unit, Medical Department, Hospital Sungai Buloh, Selangor, Malaysia; k Department of Internal Medicine, Chiang Mai University, Maharaj Nakorn Chiang Mai Hospital, Chiang Mai, Thailand; l Internal Medicine unit, Medical Department, Nakornping Hospital, Chiang Mai, Thailand; m HIV-Netherlands Australia Thailand Research Collaboration, Thai Red Cross AIDS Research Centre, Bangkok, Thailand; n Tuberculosis Research Unit, Department of Medicine, Faculty of Medicine, Chulalongkorn University, Bangkok, Thailand; o Bamrasnaradura Infectious Diseases Institute, Nonthaburi, Thailand; p Laboratory of Virology, Geneva University Hospitals, Geneva, Switzerland; q Laboratory of Virology, Program for HIV Prevention and Treatment L'Institut de Recherche pour le Développement, Chiang Mai, Thailand; r Drugs for Neglected Diseases initiative, Kuala Lumpur, Malaysia; s Drugs for Neglected Diseases initiative, New York, NY, USA; t Ministry of Health, Kuala Lumpur, Malaysia

## Abstract

**Background:**

In low-income and middle-income countries, affordable direct-acting antivirals are urgently needed to treat hepatitis C virus (HCV) infection. The combination of ravidasvir, a pangenotypic non-structural protein 5A (NS5A) inhibitor, and sofosbuvir has shown efficacy and safety in patients with chronic HCV genotype 4 infection. STORM-C-1 trial aimed to assess the efficacy and safety of ravidasvir plus sofosbuvir in a diverse population of adults chronically infected with HCV.

**Methods:**

STORM-C-1 is a two-stage, open-label, phase 2/3 single-arm clinical trial in six public academic and non-academic centres in Malaysia and four public academic and non-academic centres in Thailand. Patients with HCV with compensated cirrhosis (Metavir F4 and Child-Turcotte-Pugh class A) or without cirrhosis (Metavir F0–3) aged 18–69 years were eligible to participate, regardless of HCV genotype, HIV infection status, previous interferon-based HCV treatment, or source of HCV infection. Once daily ravidasvir (200 mg) and sofosbuvir (400 mg) were prescribed for 12 weeks for patients without cirrhosis and for 24 weeks for those with cirrhosis. The primary endpoint was sustained virological response at 12 weeks after treatment (SVR12; defined as HCV RNA <12 IU/mL in Thailand and HCV RNA <15 IU/mL in Malaysia at 12 weeks after the end of treatment). This trial is registered with ClinicalTrials.gov, number NCT02961426, and the National Medical Research Register of Malaysia, NMRR-16-747-29183.

**Findings:**

Between Sept 14, 2016, and June 5, 2017, 301 patients were enrolled in stage one of STORM-C-1. 98 (33%) patients had genotype 1a infection, 27 (9%) had genotype 1b infection, two (1%) had genotype 2 infection, 158 (52%) had genotype 3 infection, and 16 (5%) had genotype 6 infection. 81 (27%) patients had compensated cirrhosis, 90 (30%) had HIV co-infection, and 99 (33%) had received previous interferon-based treatment. The most common treatment-emergent adverse events were pyrexia (35 [12%]), cough (26 [9%]), upper respiratory tract infection (23 [8%]), and headache (20 [7%]). There were no deaths or treatment discontinuations due to serious adverse events related to study drugs. Of the 300 patients included in the full analysis set, 291 (97%; 95% CI 94–99) had SVR12. Of note, SVR12 was reported in 78 (96%) of 81 patients with cirrhosis and 153 (97%) of 158 patients with genotype 3 infection, including 51 (96%) of 53 patients with cirrhosis. There was no difference in SVR12 rates by HIV co-infection or previous interferon treatment.

**Interpretation:**

In this first stage, ravidasvir plus sofosbuvir was effective and well tolerated in this diverse adult population of patients with chronic HCV infection. Ravidasvir plus sofosbuvir has the potential to provide an additional affordable, simple, and efficacious public health tool for large-scale implementation to eliminate HCV as a cause of morbidity and mortality.

**Funding:**

National Science and Technology Development Agency, Thailand; Department of Disease Control, Ministry of Public Health, Thailand; Ministry of Health, Malaysia; UK Aid; Médecins Sans Frontières (MSF); MSF Transformational Investment Capacity; FIND; Pharmaniaga; Starr International Foundation; Foundation for Art, Research, Partnership and Education; and the Swiss Agency for Development and Cooperation.

## Introduction

About 71 million people have a chronic hepatitis C virus (HCV) infection globally, of whom approximately 399 000 die annually, mostly because of cirrhosis and hepatocellular carcinoma.^1^ The WHO Global Health Sector Strategy on Viral Hepatitis 2016–21 aims to eliminate viral hepatitis as a public health threat, test 90% of people with HCV, and treat 80% of people with HCV worldwide by 2030.^2^ However, by March, 2021, only 12 countries were on course to HCV elimination by 2030.^3^, ^4^

**Research in context**

**Evidence before this study**

In 2016, prices of direct-acting antivirals restricted access to chronic hepatitis C virus (HCV) treatment in most low-income and middle-income countries in which the burden of disease was the greatest. 50 middle-income countries, estimated to host 43% of the global HCV burden, were excluded from license agreements of originator companies for key HCV drugs, leaving a large treatment gap. Ravidasvir has been developed in a public–private partnership to be available for the treatment of HCV at an affordable price. A landscape analysis involving desk research, workshops, and interviews with key stakeholders was done by the Drugs for Neglected Diseases *initiative* in 2014, after which in-depth research into HCV was done and meetings were held with key stakeholders to evaluate the feasibility of an intervention in this area. A PubMed search was done using the terms “daclatasvir” and “ravidasvir” for clinical trials published from Jan 1, 2011, to May 2, 2016, without language restrictions, and a search was done using the same terms for abstracts from the American Association for the Study of Liver Diseases and European Association for the Study of the Liver conferences from Jan 1, 2011, to May 2, 2016. An investigator's brochure for ravidasvir was available. Before this study, there was little evidence on the efficacy of ravidasvir in patients with HCV. Efficacy of ravidasvir in combination with ritonavir-boosted danoprevir and ribavirin in patients with HCV genotype 1 had been shown in Taiwan and China, and a phase 3 study in Egypt concluded that ravidasvir plus sofosbuvir is efficacious and safe in patients with by HCV genotype 4. However, we were aware of the results of these studies, which had not yet been published, and data on the efficacy and safety of ravidasvir plus sofosbuvir in patients with other HCV genotypes were not available.

**Added value of this study**

Stage one of the STORM-C-1 study is the first trial to assess the efficacy, safety, tolerability, and pharmacokinetics of 12-week and 24-week ribavirin-free regimens of ravidasvir plus sofosbuvir in people with HCV from diverse clinical backgrounds, including patients with HIV co-infection and genotypes other than 1 or 4. Interim analysis showed that treatment in 301 patients without and with compensated cirrhosis was efficacious, regardless of HIV infection and previous interferon experience: 97% of patients had SVR12 in the full analysis set. Of note, 96% of the patients with difficult-to-cure HCV genotype 3 with cirrhosis had SVR12 without ribavirin. In this study population with high incidence of comorbidities, the regimen was well tolerated, had a favourable safety profile, and there were no clinically significant pharmacokinetic drug–drug interactions with HIV antiretrovirals commonly prescribed in Thailand and Malaysia.

**Implications of all the available evidence**

The overall high SVR12 rate in this interim analysis supports the use of ravidasvir plus sofosbuvir as a 12-week and 24-week treatment option for patients with genotype 1a, 1b, or 3 infection, either with or without compensated cirrhosis. A small number of patients with genotypes 2 and 6 were treated; the enrolment of an additional 300 patients in stage two of the study will provide additional data on efficacy and safety for these subgroups. This new regimen appears to be suitable for use in diverse populations, including patients with HIV and multiple comorbidities, and could minimise pretreatment assessments and on-treatment monitoring, allowing the management of HCV in decentralised public health settings under the supervision of appropriately trained health-care professionals. The low rate of unsuccessful treatments has potential implications for retreatment, which is important for countries in which access to salvage therapies is restricted and expensive. The use of ravidasvir plus sofosbuvir could be scaled up, together with active identification and linkage to care of patients with HCV, in test and treat approaches. It has the potential to provide an additional affordable, simple, and efficacious public health tool for large-scale implementation to eliminate HCV as a cause of morbidity and mortality in countries that do not have, or have overcome, sofosbuvir patent barriers.

Direct-acting antivirals (DAA) are an important advance in the treatment of HCV; however, only about 5 million (7%) of the 71 million patients with HCV infection received treatment with DAAs worldwide as of 2017.^5^, ^6^ Although license agreements have been signed between originator and generic companies for important HCV drugs to increase access to treatment, primarily in low-income and middle-income countries, most countries do not implement ambitious test and treat programmes because of prohibitive treatment costs.^7^, ^8^, ^9^, ^10^

Globally, the two most prevalent genotypes of HCV are genotype 1, which accounts for 44% of infections and is more prevalent in high-income countries, and genotype 3, which accounts for 25% of infections and is more prevalent in low-income and middle-income countries.^11^ Genotype 3 infection is harder to treat with DAAs, especially in patients with co-factors for poor response, such as cirrhosis and previous peginterferon-based therapy, and is associated with hepatic steatosis and increased risk of progression to cirrhosis and hepatocellular carcinoma.^12^ Regional HCV treatment guidelines differ on the treatment of genotype 3 infection: US guidelines recommend the addition of ribavirin to sofosbuvir plus velpatasvir combination regimens,^13^ whereas European guidelines do not recommend use of sofosbuvir-velpatasvir in patients with genotype 3 infection and cirrhosis, preferring recommending a longer treatment duration with glecaprevir and pibrentasvir.^14^

Ravidasvir, an oral, pangenotypic non-structural protein 5 A (NS5A) inhibitor, is under development as an affordable DAA for public health use.^15^ It has high activity against HCV genotype 3a,^16^ with inhibitory effects on HCV variants with NS5A resistance mutations.^17^ Sofosbuvir is a nucleotide analogue inhibitor of NS5B polymerase approved for treatment of HCV genotype 1–6 infections in combination with other DAAs.

At the time this study started in 2016, no safety or efficacy studies for ravidasvir had been published. Since then, three studies have been published. In 298 patients with HCV genotype 4 infection from Egypt, a 12–16 week oral formulation of ravidasvir plus sofosbuvir with or without ribavirin showed good safety and efficacy, with an overall sustained virological response at 12 weeks post-treatment (SVR12) of 95%.^18^ Ravidasvir in combination with ritonavir-boosted danoprevir and ribavirin had efficacy in patients with genotype 1 infection: 38 (100%) of 38 patients from Taiwan^19^ and 306 (96%) of 318 patients from China had SVR12.^20^ However, ribavirin is teratogenic and often poorly tolerated, necessitating additional pretreatment evaluation and more on-treatment monitoring of haemoglobin, making it unsuitable for certain subpopulations of patients with HCV.^21^

Therefore, there is a need for an affordable, pangenotypic, potent, and safe HCV treatment regimen to complement other regimens, which has low risk of drug–drug interactions and that is simple and suitable for use in decentralised public health settings. The STORM-C-1 study was designed in two stages to allow for interim analysis and presentation to relevant authorities. The aim of this study was to investigate the safety and efficacy of a ribavirin-free ravidasvir plus sofosbuvir regimen in a diverse group of patients with HCV, with or without cirrhosis.

## Methods

### Study design and participants

The first stage of this phase 2/3, multicentre, international, open-label, single-arm study was done at six public academic and non-academic centres in Malaysia and four public academic and non-academic centres in Thailand (appendix p 1). Only patients without cirrhosis were recruited in Thailand.

Patients aged 18–69 years, with a body-mass index (BMI) of 18–35 kg/m^2^, with chronic HCV infection of any genotype (viral load ≥10^4^ IU/mL) with (Metavir score of F4 with Child-Turcotte-Pugh [CTP] class A) or without (Metavir score of F0–F3) compensated liver cirrhosis, and with or without HIV co-infection were eligible for inclusion, irrespective of whether they had received a previous interferon with or without ribavirin regimen. Women of childbearing potential with a negative pregnancy test at screening and baseline; patients with virologically controlled HIV co-infection; and non-injecting drug users, including participants compliant in an opioid substitution maintenance programme, were also eligible for inclusion.

Patients with decompensated cirrhosis (evidence of advanced stage liver cirrhosis and CTP score >6 or any history of decompensation, including ascites, variceal bleeding, spontaneous bacterial peritonitis, or hepatic encephalopathy), hepatocellular carcinoma, hepatitis B virus co-infection (hepatitis B surface antigen positive), serum creatinine more than 1·5-times the upper limit of normal or end stage renal disease, and those who had received any previous NS5A inhibitor therapy, were not eligible for inclusion.

The trial was done in accordance with Good Clinical Practice Guidelines, the Declaration of Helsinki, and applicable local regulations. The initial protocol and all protocol amendments were reviewed and approved before implementation by the applicable individual institutional or national ethics committees. All patients provided written informed consent.

### Procedures

Patients without cirrhosis received 200 mg oral ravidasvir and 400 mg sofosbuvir once daily with food for 12 weeks; individuals with cirrhosis received a 24 week regimen. Regimen dose, schedule, and duration were based on all available clinical data for ravidasvir and the treatment durations used for the pharmacologically similar sofosbuvir and daclatasvir combination.

HCV RNA concentrations in serum or EDTA plasma were quantified using the COBAS AmpliPrep/COBAS TaqMan HCV Quantitative test (version 2.0; ThermoFisher Scientific, Waltham, MA, USA; lower limit of quantification 15 IU/mL) or the Abbott m2000 system (Abbott, Chicago, IL, USA; lower limit of quantification 12 IU/mL; appendix p 1).

Presence of cirrhosis was determined either by liver stiffness measurements of more than 12·5 kPa with an M probe or more than 10 kPa with an XL probe by transient elastography (FibroScan, EchoSens, Paris, France), liver biopsy, or an aspartate aminotransferase to platelet ratio index score of two or more in the absence of a liver biopsy or valid liver stiffness measurements result.

HCV genotype was determined by the Versant HCV Genotype Inno-LiPA Assay v2.0 (Siemens Healthcare Diagnostics, Tarrytown, NY, USA) in Thailand and the Abbott RealTime HCV Genotype II (Abbott Laboratories) assay in Malaysia. Genotype and subtype were confirmed using RNA sequencing of stored baseline samples, and in case of discrepancy, the sequencing derived result was used. Retrospective population sequencing (25% cutoff) of NS5A and NS5B regions was done by the Laboratory of Virology (Geneva University Hospitals, Geneva, Switzerland) or the Public Health Promotion Research and Training-Faculty of Associated Medical Sciences Laboratory (Chiang Mai University, Chiang Mai, Thailand) for all virological failures to identify pre-existing polymorphisms and characterise emerging HCV viral resistance-associated variants; any change from baseline in patients who did not have SVR12 was recorded.

Patients could withdraw from the study at any time without jeopardising their standard medical care or possible participation in future research studies. A study site investigator could decide to end a patient's participation in the study if they believed participation would be detrimental to a patient's wellbeing.

On-treatment visits were scheduled at weeks 1, 4, 8, and 12 for participants without cirrhosis. Patients with cirrhosis had additional visits at weeks 16, 20, and 24. Post-treatment visits were scheduled at weeks 4, 12, and 24 after treatment completion for all participants. Routine blood and urine test samples were collected, ECG and radiological imaging of the liver (for hepatocellular carcinoma) were done, patients were assessed for safety, and the CTP score was determined for individuals with cirrhosis according to the schedule of visits and study assessments.

To assess the possible effect of ravidasvir plus sofosbuvir treatment on concomitant antiretrovirals concentrations (tenofovir, emtricitabine, efavirenz, and nevirapine), plasma samples were collected from patients with an HIV co-infection at the same time post-dose on day 1 (ie, before the first dose of ravidasvir plus sofosbuvir), and at week 4 (ravidasvir plus sofosbuvir at steady state); and week 4 to day 1 concentration ratios for each antiretrovirals were calculated.

### Outcomes

The primary efficacy outcome was SVR12, evidenced by HCV RNA concentration less than the lower limit of quantification. The main secondary efficacy outcomes were SVR at 4 weeks (SVR4) and SVR at 24 weeks (SVR24), evidenced by HCV RNA concentration less than the lower limit of quantification; virological breakthrough, defined as either a confirmed one or higher log_10_ IU/mL increase in HCV RNA from nadir while on treatment or confirmed HCV RNA more than or equal to the lower limit of quantification if HCV RNA previously decreased to less than the lower limit of quantification while on treatment; and virological relapse, defined as HCV RNA less than the lower limit of quantification at the end of treatment but more than or equal to the lower limit of quantification during the post-treatment period. Reinfections (patients with different HCV genotype at baseline and relapse) were not considered virological failures. Non-virological failure was any failure that did not meet the virological failure criteria (appendix pp 2–3).

Safety outcomes included occurrence of the following events until 24 weeks after the end of treatment: any adverse event, treatment-related adverse event, adverse event leading to premature treatment discontinuation, laboratory abnormality, grade 3 or 4 adverse event, serious adverse event, or death.

Adverse events and severe adverse events were graded according to the National Institutes of Health and National Institute of Allergy and Infectious Disease Division of Acquired Immune Deficiency Syndrome Table for Grading the Severity of Adult and Paediatric Adverse Events (version 2.0). Treatment-emergent adverse events were defined as adverse events that started between the date of first study drug dose and the end of study visit, or before the date of first study drug dose but worsened between the date of first study drug dose and the end of study visit. Safety endpoints were assessed by the principal investigator (IA-M and S-ST) and by representatives (VG, CM, FS, SS) of Drugs for Neglected Diseases *initiative* (DND*i*). Relatedness of adverse events to the study drugs was assessed by the principal investigators (S-ST, HR, MR, HO, HT, WKC, SK, ST, KT, AA, and SK). Adverse events were assessed by representatives from the DND*i* only if they were serious. The sponsor assessed safety endpoints as part of the statistical analysis. In line with clinical trial practices, the sponsor assessed each serious adverse events for seriousness, relatedness, and expectedness to determine if the serious adverse event is reportable in an expedited manner to health authorities or other investigators.

The ratios of week 4 to day 1 antiretroviral plasma concentrations were calculated. 25 patients in Malaysia underwent intensive blood sampling to assess ravidasvir pharmacokinetics.

Baseline factors associated with SVR12 outcome and changes in HCV NS5A sequences from treatment initiation in those who did not have a SVR12 response were also assessed as a secondary endpoint. Changes in the PROQOL-HCV domain scores and time to premature treatment and study discontinuation, to first treatment-emergent adverse events, to first grade 3–4 treatment-emergent adverse events, and to first treatment-emergent severe adverse events are reported in the appendix (pp 11–16).

### Statistical analysis

The interim analysis of the efficacy, safety, tolerance, and pharmacokinetics of ravidasvir plus sofosbuvir was planned when 300 patients had completed or prematurely discontinued the study. We calculated that this sample size would provide more than 86% power to detect 6% or higher improvement in the overall SVR12 rate from a prespecified performance goal of 85% (two-sided exact binomial test, α=0·05). This goal was based on published results for other treatments and in line with a WHO publication describing the ideal public health HCV treatment.^22^, ^23^, ^24^, ^25^, ^26^ On the basis of results of previous clinical trials evaluating the efficacy of ravidasvir, we estimated that the overall SVR12 rate would be at least 91% (ie, at least 6% more than the prespecified performance goal). Proceeding to stage two of the study would only occur if the efficacy and safety results from stage one were considered satisfactory by the Data and Safety Monitoring Board, specifically if the lower bound of the 95% CI for the observed overall SVR12 rate was more than 85% in the intention to treat analysis and there were no safety issues (deaths or grade 3 or 4 treatment-related treatment-emergent adverse events). Stage two will supplement these interim results with additional information on the performance of ravidasvir plus sofosbuvir against the main HCV genotypes found in Malaysia and Thailand (genotype 1, 3, 6, and to a lesser extent 2). The final analysis is planned in 600 patients, combining data from both stages. This sample size would ensure that the width of the 95% CI for the expected overall SVR12 rate would be lower than 5%. A separate analysis of the stage two results is not planned.

As planned in the protocol, the efficacy of ravidasvir plus sofosbuvir was evaluated overall and in each of the following prespecified subgroups: patients with or without cirrhosis, each HCV genotype, patients who had previous or no previous interferon therapy, and those with or without HIV co-infection. The study was not powered for a subgroup analysis and these results are presented using descriptive statistics.

Primary analysis of efficacy outcomes was done for all patients who received at least one dose of study drug and had not reported current active injection drug use at screening (full analysis set). A secondary analysis (per-protocol set) included all patients in the full analysis set population who did not prematurely discontinue the study, who had a 90% or higher adherence to ravidasvir plus sofosbuvir (number of tablets used divided by number of tablets prescribed), did not have missing SVR12 results, and had no major protocol deviations, such as eligibility criteria not met, during the study. If a post-treatment HCV RNA value was missing within the analysis window and was followed by a valid value, the missing value was substituted with the subsequent valid value. The 95% CI for proportions were calculated using the exact Clopper-Pearson method. Analysis of safety outcomes was done in all enrolled patients who received at least one dose of a study drug.

Statistical analyses were done using Stata (version 14.1). Two independent adjudication committees determined whether HCV viral load values were evaluable and adjudicated key data used for the analysis. Efficacy and safety results were reviewed by an independent Data and Safety Monitoring Board. This trial is registered with ClinicalTrials.gov, number NCT02961426, and the National Medical Research Register of Malaysia, NMRR-16-747-29183.

### Role of the funding source

The study was designed and done by the investigators and the sponsor, the DND*i*. Study analysis and financial support were provided by the DND*i*. The DND*i* participated in the interpretation of data and the review and approval of the Article. The National Science and Technology Development Agency, Thailand and Department of Disease Control, Ministry of Public Health, Thailand were responsible for funding the study in Thailand and the Ministry of Health, Malaysia was responsible for financing the study in Malaysia.

## Results

**Figure 1:**
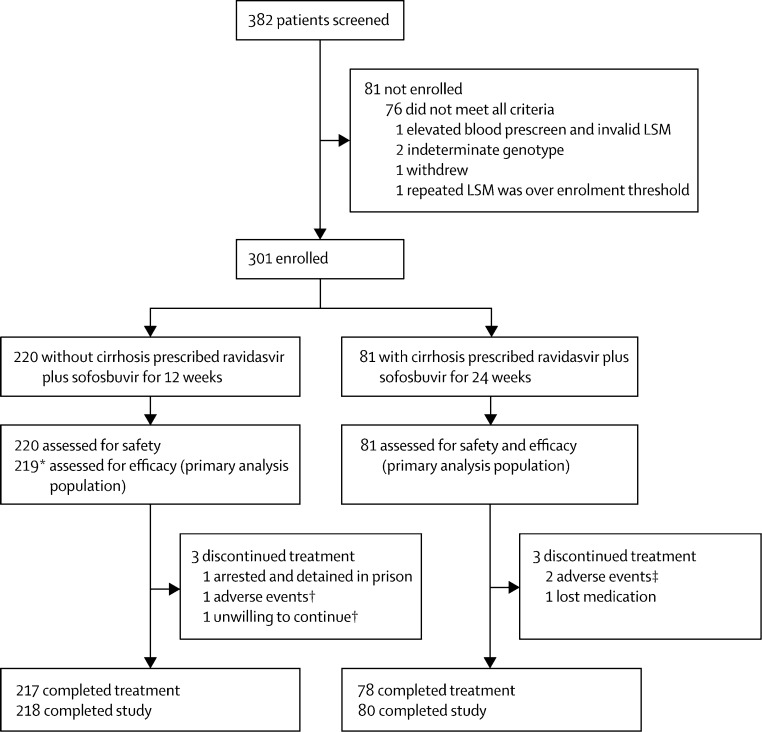
Trial profile LSM=liver stiffness measurement. *One patient with active injection drug use at eligibility visit was excluded from the full analysis set. † Patient did not complete study. ‡One patient with adverse events discontinued treatment and did not complete the study.

382 patients were screened between Sept 14, 2016, and June 5, 2017. 81 (21%) patients were excluded after screening (figure 1). 301 patients were enrolled onto the study, of whom 231 (77%) were men, 158 (52%) had genotype 3 infection, 99 (33%) had been previously treated with interferon regimens with or without ribavirin, and 90 (30%) had a HIV co-infection (table 1). The median age was 47 years (IQR 40–55). 133 (44%) patients had a history of injection drug use, and 25 (8%) were receiving methadone at baseline. Median liver stiffness measurements scores were 6·9 kPa (IQR 5·5–8·9) for the 220 (73%) patients without cirrhosis and 21·3 kPa (16·9–27·7) for the 81 (27%) patients with cirrhosis. 215 (71%) patients had baseline HCV RNA of 800 000 IU/mL or more.

**Table 1:** Demographic and baseline characteristics

		**12 weeks ravidasvir plus sofosbuvir (n=220)**	**24 weeks ravidasvir plus sofosbuvir (n=81)**	**Overall (n=301)**
Sex				
	Men	170 (77%)	61 (75%)	231 (77%)
	Women	50 (23%)	20 (25%)	70 (23%)
Age groups (years)				
	<50	143 (65%)	31 (38%)	174 (58%)
	≥50 to <65	75 (34%)	46 (57%)	121 (40%)
	≥65	2 (1%)	4 (5%)	6 (2%)
Age (years)				
	Mean (SD)	44·1 (10·9)	51·4 (8·6)	46·1 (10.8)
	Median (IQR)	44 (37–52)	53 (46–57)	47 (40–55)
	Range	20–66	25–67	20–67
Country of treatment				
	Malaysia	139 (63%)	81 (100%)	220 (73%)
	Thailand	81 (37%)	0	81 (27%)
HCV genotype				
	1a	76 (35%)	22 (27%)	98 (33%)
	1b	21 (10%)	6 (7%)	27 (9%)
	2	2 (1%)	0	2 (1%)
	3	105 (48%)	53 (65%)	158 (52%)
	6	16 (7%)	0	16 (5%)
HCV RNA (IU/mL)				
	<800 000	61 (28%)	24 (30%)	85 (28%)
	≥800 000	158 (72%)	57 (70%)	215 (71%)
	Missing	1 (<1%)	0	1 (<1%)
HIV and HCV co-infection		79 (36%)	11 (14%)	90 (30%)
Previous interferon exposure		67 (30%)	32 (40%)	99 (33%)
Reported injection drug use				
	Current	1 (<1%)	0	1 (<1%)
	Past	99 (45%)	34 (42%)	133 (44%)
	None	120 (55%)	47 (58%)	167 (55%)
Median liver stiffness (kPa)		6·9 (5·5–8·9)	21·3 (16·9–27·7)	8·6 (6·1–14·0)
Use of concomitant medication		162 (74%)	67 (83%)	229 (76%)
Common comorbidities*				
	Hypertension	35 (16%)	30 (37%)	67 (22%)
	Diabetes mellitus	18 (8%)	24 (30%)	42 (14%)
	Non-alcoholic fatty liver	15 (7%)	4 (5%)	19 (6%)
	Obesity	9 (45)	9 (11%)	18 (6%)
	Dyslipidaemia	11 (5%)	5 (6%)	16 (5%)
Interleukin 28 genotype				
	Cytosine, cytosine	162 (74%)	60 (74%)	222 (74%)
	Cytosine, thymidine	58 (26%)	20 (25%)	78 (26%)
	Thymidine, thymidine	0	1 (1%)	1 (<1%)

Data are n (%) unless otherwise stated. Data are for the safety analysis population. HCV=hepatitis C virus.

* In at least 5% of patients.

All enrolled patients received at least one dose of study drug and were included in the safety analysis set. No patients were lost to follow-up, but six (2%) discontinued treatment (figure 1). Three of these six treatment discontinuations were due to treatment-emergent adverse events: one patient with cirrhosis, with genotype 3, without previous interferon therapy, and without HIV co-infection stopped treatment 9 days after initiation because of tiredness, palpitations, hot flush, and breast engorgement and discontinued the study 2 days later; one patient with cirrhosis, with genotype 1a, with previous interferon therapy, and with HIV co-infection stopped treatment 2 days after initiation because of vomiting, palpitations, and transient mild prolonged QT; and one patient without cirrhosis, with genotype 3, without previous interferon therapy, and with HIV co-infection stopped treatment 1 day after initiation due to fatigue, diarrhoea, and abdominal discomfort and discontinued the study 28 days later.

Seven enrolled patients (three [1%] without cirrhosis and four [5%] with cirrhosis) had major protocol deviations (per-protocol set, not included in figure 1; appendix p 6). One patient who reported use of intravenous illicit drugs was enrolled; however, their data contributed only to the safety analysis set.

**Figure 2:**
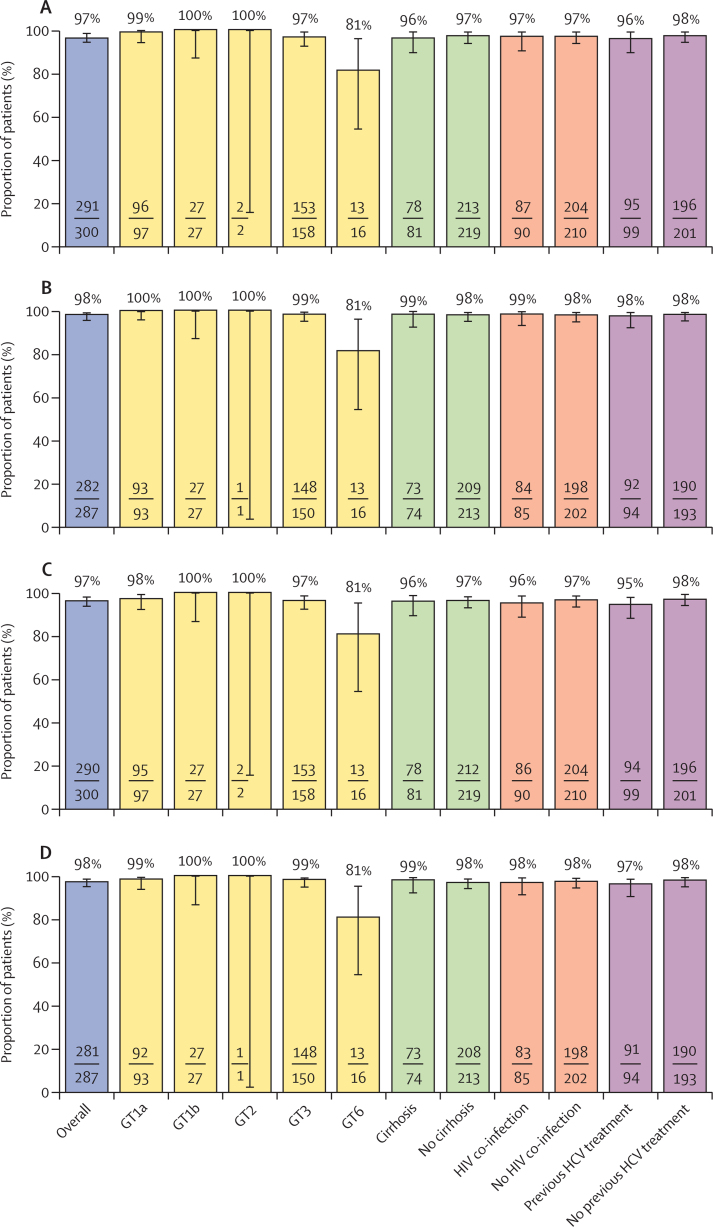
SVR12 and SVR24 in key patient subgroups SVR12 in the full analysis set (A) and the per-protocol set (B). SVR24 in the full analysis set (C) and the per-protocol set (D). Error bars are 95% CI. GT=genotype. SVR12=sustained virological response 12 weeks after end of treatment. SVR24=sustained virological response 24 weeks after the end of treatment.

291 (97%, 95% CI 94–99) of 300 patients in the full analysis set had SVR12, significantly higher than the prespecified performance goal of 85% (p<0·0001; figure 2; appendix pp 6–7). SVR12 results higher than 95% were observed in key subpopulations, including 78 (96%) of 81 patients with cirrhosis, 87 (97%) of 90 patients with an HIV co-infection, 95 (96%) of 99 patients with previous interferon experience, and in patients with HCV genotypes 1a (96 [99%] of 97 patients), 1b (27 [100%] of 27), and 3a (153 [97%] of 158). Of note, SVR12 was reported in 133 (96%) of 138 patients (85 [97%] of 88 without cirrhosis and 48 [96%] of 50 with cirrhosis) with genotype 3a and by all 17 patients (14 without cirrhosis and three with cirrhosis) with genotype 3b (three patients had unknown genotype 3 subtype). Patients with genotype 6 infection had the lowest rate of SVR12 (13 [81%] of 16 patients). Both patients with genotype 2 infection had SVR12. There was no difference in SVR12 rates by sex, age, ethnicity, country, cirrhotic status, interleukin 28B gene polymorphism, previous interferon regimen with or without ribavirin, HIV co-infection status, baseline BMI, or baseline HCV RNA, alanine aminotransferase, or aspartate aminotransferase concentration in the full analysis set (appendix pp 6–7). SVR12 rates by combination of key subgroups are provided in table 2. In the per-protocol population, 282 (98%, 95% CI 96–99) of 287 patients had SVR12.

**Table 2:** SVR12 by combination of key subgroups

	**Genotype 1a**	**Genotype 1b**	**Genotype 2**	**Genotype 3**	**Genotype 6**
**Patients with no cirrhosis but with HIV coinfection**					
Previous interferon-based treatment	10/10 (100%)	3/3 (100%)	NA	6/6 (100%)	1/1 (100%)
No previous interferon-based treatment	25/25 (100%)	3/3 (100%)	1/1 (100%)	21/22 (95%)	7/8 (88%)
**Patients with no cirrhosis and no HIV co-infection**					
Previous interferon-based treatment	12/12 (100%)	4/4 (100%)	1/1 (100%)	28/29 (97%)	0/1 (0%)
No previous interferon-based treatment	28/28 (100%)	11/11 (100%)	NA	47/48 (98%)	5/6 (83%)
**Patients with cirrhosis and HIV co-infection**					
Previous interferon-based treatment	1/2 (50%)	NA	NA	2/2 (100%)	NA
No previous interferon-based treatment	4/4 (100%)	1/1 (100%)	NA	2/2 (100%)	NA
**Patients with cirrhosis but no HIV co-infection**					
Previous interferon-based treatment	4/4 (100%)	3/3 (100%)	NA	20/21 (95%)	NA
No previous interferon-based treatment	12/12 (100%)	2/2 (100%)	NA	27/28 (96%)	NA

Data are n/N (%). Data are for the final analysis set. SVR12=sustained virological response 12 weeks after end of treatment. NA=not applicable.

In the full analysis set, 290 (97%; 95% CI 94–98) of 300 patients had SVR24, with no noticeable differences between subgroups (figure 2). There was high concordance between SVR12 and SVR24: only one patient with SVR12 did not have SVR24. In the full analysis set, concordance between SVR4 and SVR12 was seen in 291 (97%) of 293 participants. Two individuals (1%) had SVR4 and but not SVR12. In the per-protocol population, 119 (99%) of 120 patients with genotype 1 infection and 148 (99%) of 150 patients with genotype 3 infection had SVR24. 13 (81%) of 16 patients with genotype 6 infection had SVR24. Both patients without cirrhosis with genotype 2 infection had SVR24.

In the full analysis population, the mean HCV RNA count was 6·15 log_10_ IU/mL (SD 0·82) at baseline. The proportion of patients with HCV RNA less than the lower limit of quantification at each on-treatment visit is presented in table 3.

**Table 3:** Proportion of patients with cirrhosis and without cirrhosis with HCV RNA less than LLOQ by on-treatment visit

	**Patients without cirrhosis with HCV RNA less than LLOQ**		**Patients with cirrhosis with HCV RNA less than LLOQ**		**Overall**	
	Proportion of patients	95% CI	Proportion of patients	95% CI	Proportion of patients	95% CI
Week 1	40/215 (19%)	14–25%	11/80 (14%)	7–23%	51/295 (17%)	13–22%
Week 4	196/215 (91%)	87–95%	71/78 (91%)	82–96%	267/293 (91%)	87–94%
Week 8	211/215 (98%)	95–99%	76/79 (96%)	89–99%	287/294 (98%)	95– 99%
Week 12	212/212 (100%)	98–100%	78/79 (99%)	93–100%	290/291 (>99%)	98–100%
Week 16	NA*	NA*	79/79 (100%)	95–100%	79/79 (100%)	95–100%
Week 20	NA*	NA*	78/79 (99%)	93–100%	78/79 (99%)	93–100%
Week 24	NA*	NA*	77/78 (99%)	93–100%	77/78 (99%)	93–100%

Data are n/N (%), unless otherwise specified. Data are for the final analysis set. HCV=hepatitis C virus. LLOQ=lower limit of quantification (defined as HCV RNA by PCR <12 IU/mL or <15 IU/mL). NA=not applicable.

* NA because participants received treatment for only 12 weeks.

Nine (3%) of 300 patients (three with cirrhosis and six without cirrhosis) in the full analysis set did not have SVR12 (table 4). The accumulation of risk factors (cirrhosis, HIV co-infection, previous HCV treatment, and HCV genotype) did not significantly affect SVR12 rates (table 2).

**Table 4:** Reasons for unsuccessful treatment at follow-up week 12 by key subgroups

		**Cirrhosis**		**Genotype 3**		**HIV co-infection**		**HCV prior treatment**		**Overall (n=9)**
		No (n=6)	Yes (n=3)	Yes (n=5)	No (n=4)	Yes (n=3)	No (n=6)	Yes (n=4)	No (n=5)	
Virological failure		4 (67%)	1 (33%)	2 (40%)	3 (75%)	1 (33%)	4 (67%)	2 (50%)	3 (60%)	5 (56%)
	Virological breakthrough*	0	1/1 (100%)	1/2 (50%)	0	0	1/4 (25%)	1/2 (50%)	0	1/5 (20%)
	Virological relapse†	4/4 (100%)	0	1/2 (50%)	3/3 (100%)	1/1 (100%)	3/4 (75%)	1/2 (50%)	3/3 (100%)	4/5 (80%)
Non-virological failure		2 (33%)	2 (67%)	3 (60%)	1 (25%)	2 (67%)	2 (33%)	2 (50%)	2 (40%)	4 (44%)

Data are n/N (%). HCV=hepatitis C virus. LLOQ=lower limit of quantification.

* Defined as either confirmed 1 log_10_ IU/mL or higher increase in HCV RNA from nadir while on treatment, or confirmed HCV RNA more than LLOQ if HCV RNA previously declined to less than LLOQ while on treatment.

† Defined as HCV RNA less than LLOQ at the end of treatment but HCV RNA equal to or more than LLOQ during the post treatment period.

The distribution of NS5A resistance-associated variants detected in 91 (31%) of 292 patients at baseline was heterogeneous per genotype (appendix p 7). All 201 patients without baseline NS5A resistance-associated variants and 86 (95%) of 91 patients with one or more baseline NS5A resistance-associated variants had SVR12. No new NS5A resistance-associated variants emerged in any patient who had virological failure. One patient had an emergent NS5B (Cys282Thr) resistance-associated variant after virological failure. Patients with genotype 1, 2, or 3b infection with single or multiple NS5A resistance-associated variants all had SVR12; data for SVR12 by genotype in all patients with resistance-associated variants are shown in the appendix (p 7). 21 (91%) of 23 patients with HCV genotype 3a and the 93His NS5A resistance-associated variant had SVR12. 11 (69%) of 16 patients with genotype 6 infection, including all three patients with virological failure, had the double NS5A resistance-associated variants (28Val and 93Ser). Ten (91%) of these 11 patients (including two of the three patients with virological failure) had genotype 6n infection. Of the 158 study participants with genotype 3 infection, two (1%) patients had virological failure. One patient with liver cirrhosis who had received previous interferon-based therapy had virological breakthrough at week 20 and had 93His at baseline and at failure. The other patient was interferon naive and did not have cirrhosis; this patient relapsed at 12 weeks after end of treatment and had a mix of both 93Tyr and 93His at baseline and 93His at failure. No NS5B resistance-associated variants were observed at baseline or at failure for either patient.

The most common treatment-emergent adverse events were pyrexia (35 [12%] of 301), cough (26 [9%]), upper respiratory tract infection (23 [8%]), and headache (20 [7%]; table 5). Grade 3 or worse adverse events were reported by 21 (7%) patients. There was no clinically meaningful difference in the frequency of adverse events by cirrhosis status (table 5), nor by HIV status and genotype subgroups (data not shown).

**Table 5:** Overview of treatment emergent adverse events

		**12 weeks ravidasvir plus sofosbuvir (n=220)**	**24 weeks ravidasvir plus sofosbuvir (n=81)**	**Overall (n=301)**
Any treatment-emergent adverse event		136 (62%)	56 (69%)	192 (64%)
	Any treatment-emergent adverse event resulting in death	0	0	0
	Any treatment-emergent adverse event of grade 3 or worse	13 (6%)	8 (10%)	21 (7%)
	Any treatment-emergent serious adverse event	11 (5%)	8 (10%)	19 (6%)
	Any treatment-emergent adverse event leading to permanent treatment discontinuation	1 (<1%)	2 (2%)	3 (1%)
Any treatment-related treatment-emergent adverse event		62 (28%)	25 (31%)	87 (29%)
	Any treatment-related treatment-emergent adverse event of grade 3 or worse	2 (1%)	1 (1%)	3 (1%)
	Any treatment-related treatment-emergent serious adverse event	1 (<1%)	0	1 (<1%)
	Any treatment-related treatment-emergent adverse event leading to permanent treatment discontinuation	1 (<1%)	1 (1%)	2 (1%)
Most common treatment-emergent adverse events (in 5% or more patients)				
	Pyrexia	25 (11%)	10 (12%)	35 (12%)
	Cough	19 (9%)	7 (9%)	26 (9%)
	Upper respiratory tract infection	19 (9%)	4 (5%)	23 (8%)
	Headache	14 (6%)	6 (7%)	20 (7%)
	Dizziness	13 (6%)	4 (5%)	17 (6%)
	Rhinorrhoea	15 (7%)	4 (5%)	19 (6%)
	Lethargy	9 (4%)	7 (9%)	16 (5%)
	Nausea	12 (5%)	3 (4%)	15 (5%)
	Diarrhoea	12 (5%)	2 (2%)	14 (5%)
	Hypertension	7 (3%)	7 (9%)	14 (5%)
ALT, AST, and bilirubin results of grade 3 and higher		2 (1%)	2 (2%)	4 (1%)
	AST elevated	0	2 (2%)	2 (1%)
	ALT elevated	0	0	0
	Bilirubin conjugated increased	2 (1%)	0	2 (1%)

Data are n (%). Data are for the safety analysis set. Patients with more than one treatment-emergent adverse or severe adverse event were counted once according to their highest graded event. There was no increase in International Normalized Ratio of grade 3 or above. Adverse events were coded using the Medical Dictionary for Regulatory Activities (version 20.0). AST=aspartate aminotransferase. ALT=alanine aminotransferase.

24 treatment-emergent serious adverse events occurred in 19 (1%) of 301 patients in the safety analysis set (appendix pp 9–10). Of the nine treatment-emergent serious adverse events that occurred on treatment, three (33%) were classified as grade 1 or 2 and six (67%) were grade 3. Of the 15 treatment-emergent serious adverse events that occurred post-treatment, four (27%) were classified as grade 1 or 2 and 11 (73%) as grade 3.

All serious adverse events were judged as not related to ravidasvir plus sofosbuvir treatment by both investigator and sponsor, except for one patient with transient acute kidney injury, which was assessed as possibly related to sofosbuvir by the investigator. There were no treatment discontinuations due to serious adverse events related to study drugs. One serious adverse event assessed as not related to the study drug led to permanent treatment discontinuation. In three (1%) of 301 patients there were ten treatment-emergent adverse events leading to treatment discontinuation. Five of these events were considered to be treatment-related (tiredness, palpitations, hot flush, breast engorgement, and fatigue), two were considered probably related (diarrhoea and abdominal discomfort), and three events considered not related (vomiting, investigations of cardiac disorders, and lethargy).

Mean adherence to the study drugs in the full analysis set was 98% (SD 11), with treatment compliance rates similar between patients without cirrhosis (99% [10]) and with cirrhosis (97% [15]). There were no clinically relevant vital sign changes or physical findings. There were no grade 4 treatment-emergent laboratory abnormalities. Bilirubin concentrations were elevated in 30 (10%) of 301 patients (16 [7%] of 220 patients without cirrhosis and 14 [17%] of 81 patients with cirrhosis). Four hyperbilirubinaemia events were classed as grade 3, none of which were considered clinically significant. There was no signal of drug-induced liver toxicity. None of the patients had a change in ravidasvir or sofosbuvir dose during the study.

In the subset of patients with HIV co-infection receiving efavirenz, tenofovir disoproxil fumarate, emtricitabine, or nevirapine, concomitant treatment with ravidasvir plus sofosbuvir did not affect serum creatinine concentration, estimated glomerular filtration rate, HIV viral suppression, or CD4 cell counts. Of 63 patients receiving tenofovir disoproxil fumarate-based antiretrovirals, mean creatinine clearance by Cockcroft-Gault equation remained stable from baseline (95·9 mL/min [SD 27·1]) to 12 weeks (94·1 mL/min [26·8]) in patients without cirrhosis and from baseline (93·6 mL/min [41·4]) to 24 weeks (99·8 mL/min [47·3]) in patients with cirrhosis. Measurement of antiretrovirals plasma concentrations revealed no clinically significant changes in antiretroviral concentrations at week 4 compared with the day before ravidasvir plus sofosbuvir treatment initiation and no antiretroviral dose adjustments were needed (appendix p 10). For ravidasvir, the mean ravidasvir AUC_0–24_was 19·92 (SD 12·77) hr·μg/mL, mean C_max_ was 2·54 (SD 1·21) μg/mL, and mean C_last_ was 0·19 (SD 0·20) μg/mL. Pharmacokinetic and pharmacodynamic data for ravidasvir are reported in the appendix (pp 4–5).

## Discussion

Interim analysis of results for the 301 patients enrolled in stage one of the STORM-C-1 study show that 12 or 24 weeks of treatment with ravidasvir plus sofosbuvir was efficacious in those with HCV genotype 1a, 1b, or 3 infection, either with and without cirrhosis. In the full analysis set, 97% patients had SVR12 and SVR24. Similar SVR12 and SVR24 rates were observed consistently across subgroups, except for a lower SVR12 rate (81%) in the small number of patients with genotype 6 infection. The low number of patients makes it difficult to draw conclusions for genotypes 2 and 6; as a result, more patients with these genotypes have been recruited in stage two of STORM-C-1 to evaluate ravidasvir plus sofosbuvir efficacy more accurately in these populations.

The high SVR12 rate in patients with genotype 3 infection and cirrhosis is particularly relevant for resource-limited settings, in which retreatments for unsuccessful DAA regimens are unavailable or unaffordable. The SVR12 rate in this study is higher than that reported in the ALLY study^24^ (20 [63%] of 32 patients), which assessed a 12-week regimen of sofosbuvir plus daclatasvir,^24^ the French Temporary Authorisation for Use programme with sofosbuvir plus daclatasvir for 12-weeks (31 [67%] of 46 patients) or 24-weeks (185 [95%] of 195 patients),^23^ the ASTRAL-3 study^27^ of a 12-week regimen of sofosbuvir plus velpatasvir (73 [91%] of 80 patients), and an a study of a 12-week regimen of sofosbuvir plus velpatasvir (seven [50%] of 14 patients with GT3b HCV).^28^ This ravidasvir plus sofosbuvir regimen might be appropriate for countries with high prevalence of genotype 3, seeking a ribavirin-free treatment algorithm that will have efficacy in most patients and can be effectively scaled up, without burdening over-stretched health-care systems or compromising response rates. The rapid suppression of HCV RNA after treatment initiation might be useful for prevention of forward transmission in certain clinical settings and populations engaged in high-risk behaviours, such as injection drug use, tattooing, men having sex with men, and sex workers. As in other trials, we observed high concordance between SVR12 and SVR24 (97%).^29^

There were no deaths and a small number of treatment-emergent severe adverse events during and after treatment. Additionally, there was no safety signal and no need for dose adaptation during the study treatment. No patients were withdrawn from the study because of treatment-emergent severe adverse events related to study drugs.

Drug–drug interactions between antiretrovirals and daclatasvir and velpatasvir necessitate either switching antiretrovirals or adjusting the dose of daclatasvir. In participants with HIV, no clinically significant drug–drug interactions between ravidasvir plus sofosbuvir and antiretrovirals commonly used in this region were found, and no antiretroviral dose adjustments were needed. None of the patients had a change in ravidasvir or sofosbuvir dose during the study. The treatment was well tolerated, overall compliance rates were consistently high throughout the study and in all groups (>95% for both study drugs), and the safety profile appears similar to that observed for other NS5A inhibitor DAAs in terms of frequency, severity, seriousness, and adverse events leading to treatment discontinuation.^24^, ^27^, ^30^ Combining ravidasvir plus sofosbuvir with tenofovir disoproxil fumarate did not affect serum creatinine concentration or creatinine clearance (calculated with the Cockcroft-Gault equation) during or after treatment. The safety profile, high rates of treatment compliance, and absence of clinically relevant pharmacological interactions with commonly used antiretrovirals indicate the potential for effective use of this combination in primary care settings with a small amount of monitoring required and without the need for complex adjustment of co-medications.

Ravidasvir plus sofosbuvir is efficacious in the presence of baseline resistance-associated variants, and no new NS5A resistance-associated variants were seen in the small number of unsuccessful treatments.

This study was designed to reflect real-world patient populations with high percentages of comorbidities and co-medications. Because few exclusion criteria prevented enrolment, the study population is representative of the general population of patients with HCV in Thailand and Malaysia. The recruited population showed a high rate of compliance, potentially indicating a recruitment bias leading to overestimation of efficacy. The study was done in highly skilled centres to ensure Good Clinical Practice compliance and optimal patient care, potentially introducing selection bias by recruiting more difficult to treat patients, leading to underestimation. Given the small number of patients in each subgroup, firm conclusions cannot be made about efficacy in patients with genotype 2 or genotype 6 infection. The results cannot be generalised to patients with HCV and decompensated cirrhosis or hepatitis B co-infection because decompensated cirrhosis or hepatitis B co-infection were excluded in this study protocol. Because no control group was used, the efficacy of sofosbuvir and ravidasvir cannot be formally compared with other DAAs.

This easy to use, ribavirin-free regimen is well tolerated, has no clinically relevant drug–drug interactions, and does not require intensive pretreatment evaluation or on-treatment monitoring. The SVR12 rate is high in otherwise difficult to treat patients with genotype 3 infection and cirrhosis, the effect of baseline resistance-associated variants is small, and few patients would need retreatment. This ravidasvir plus sofosbuvir combination appears suitable for use in diverse populations, including individuals with HIV and multiple other comorbidities, and it could provide an additional affordable public health solution with some key advantages for countries that do not have, or have not overcome, sofosbuvir patent barriers. This regimen might be useful in decentralised public health settings in countries, such as Thailand and Malaysia, in which a high proportion of the population are estimated to have a chronic HCV infection.^11^ The use of ravidasvir plus sofosbuvir could be scaled up, together with active identification and linkage to care of HCV infected persons, in test and treat approaches. An ongoing second stage with 300 additional patients will provide more information on the performance of ravidasvir plus sofosbuvir in the main genotypes found in Malaysia and Thailand.

## Data sharing

The data underlying the results of this study are available upon request because they contain potentially sensitive information. Interested researchers can contact the Drugs for Neglected Diseases initiative, the commissioner of this study, for data access requests via email (CTdata@dndi.org). Researchers can also request data by completing the form available online. In this, they confirm that they will share data and results with DND*i* and will publish any results under an open access license.

**This online publication has been corrected. The corrected version first appeared at thelancet.com/gastrohep on July 6, 2022**
