# Supplementary material for: Efficacy and safety of ravidasvir plus sofosbuvir in patients with chronic hepatitis C infection without cirrhosis or with compensated cirrhosis (STORM-C-1): interim analysis of a two-stage, open-label, multicentre, single arm, phase 2/3 trial
Source: Lancet Gastroenterol Hepatol. 2021 Apr 16;6(6):448–58. doi: 10.1016/S2468-1253(21)00031-5 (PMC9767645; doi:10.1016/S2468-1253(21)00031-5)
Supplement: Supplementary appendix [file mmc1.pdf]

THE LANCET
Gastroenterology & Hepatology

Supplementary appendix

This appendix formed part of the original submission and has been peer reviewed. We post it as supplied by the authors.

Supplement to: Andrieux-Meyer I, Tan S-S, Thanprasertsuk S, et al. Efficacy and safety of ravidasvir plus sofosbuvir in patients with chronic hepatitis C infection without cirrhosis or with compensated cirrhosis (STORM-C-1): interim analysis of a two-stage, open-label, multicentre, single arm, phase 2/3 trial. *Lancet Gastroenterol Hepatol* 2021; published online April 15. https://doi.org/10.1016/S2468-1253(21)00031-5.

Appendix: Efficacy and safety of ravidasvir plus sofosbuvir in subjects with chronic hepatitis C infection with and without compensated cirrhosis: interim results of an open-label, multicentre, single arm phase 2/3 trial

List of study sites for the STORM-C-1 study with Principal Investigators and number of patients recruited

Site Number	Study Site	Country	Site Principal Investigator	Number of patients recruited, N=301
03	Hospital Ampang	Malaysia	Dr. Hajjah Rosaida Hj Mohd Said.	47
04	Hospital Sultanah Bahiyah	Malaysia	Datuk Dr. Muhammad Radzi Abu Hassan	46
05	Hospital Tengku Ampuan Afzan	Malaysia	Dr. Tee Hoi Poh	42
01	Hospital Selayang	Malaysia	Dr. Haniza Omar	42
06	University Malaya Medical Centre	Malaysia	Assoc. Prof. Chan Wah Kheong	23
09	Bamrasnaradura Infectious Diseases Institute,	Thailand	Suparat Khemnark, MD,	21
02	Hospital Sungai Buloh	Malaysia	Dr. Suresh Kumar	20
07	Maharaj Nakorn, Chiang Mai Hospital	Thailand	Assoc. Prof. Satawat Thongsawat, MD,	20
08	Nakornping Hospital	Thailand	Kanawee Thetket, MD,	20
10	King Chulalongkorn Memorial Hospital/HIV-NAT, Bangkok	Thailand	Anchalee Avihingsanon, MD	20

Methods for quantification of HCV RNA in serum or EDTA plasma for the STORM-C-1 study

Country	Laboratory	Machine used for Real-Time PCR	Lower limit of quantification	Sites	Visits
Malaysia	Hospital Kuala Lumpur	Roche COBAS® AmpliPrep & COBAS® TaqMan®48 system	15 IU/mL	All sites in Malaysia	All visits
Thailand	HIVNAT AIDS Research Centre	Abbott M2000 system	12 IU/mL	Site 10	Screening visit only
	Ramathibodi Hospital	Roche COBAS® AmpliPrep/COBAS® TaqMan® system	15 IU/mL	Site 9	Screening visit only
	AMS Clinical Service Center	Roche COBAS® AmpliPrep/COBAS® TaqMan® system	15 IU/mL	Sites 7 & 8	Screening visit only
	IRD-PHPT Research Group	Abbott M2000 system	12 IU/mL	All sites in Thailand	All visits except screening visit

Full list of objectives and outcomes for the STORM-C-1 study

Study Objectives		Study Endpoints	
Primary objective	To assess the efficacy of 12 weeks (RDV-SOF) in subjects with chronic HCV infection and no cirrhosis (Metavir F0 to F3), and 24 weeks SOF-RDV in subjects with compensated cirrhosis (Metavir F4 and CTP class A), 12 weeks after the end of study treatment.	Primary endpoint	SVR12, as evidenced by HCV RNA level less than the LLOQ.
Secondary objective	To assess the efficacy of 12 weeks RDV-SOF in subjects with chronic HCV infection and no cirrhosis and 24 weeks RDV-SOF in subjects with compensated cirrhosis at 4 and 24 weeks after the end of study treatment.	Secondary endpoints	Sustained virologic response at 4 and 24 weeks post treatment completion (SVR4 and SVR24), as evidenced by HCV RNA level less than the lower limit of quantification.<br>**Among subjects not achieving SVR12:**<br>Occurrence of on-treatment virologic failure, defined as HCV RNA ≥ LLOQ at the end of the treatment period.<br>Occurrence of virologic breakthrough, defined as either confirmed ≥ 1 log10 IU/mL increase in HCV RNA from nadir while on treatment or confirmed HCV RNA ≥ LLOQ if HCV RNA previously declined to < LLOQ while on treatment.<br>Occurrence of virologic relapse, defined as HCV RNA < LLOQ at the end of the treatment period but HCV RNA ≥ LLOQ during the post-treatment period.<br>Occurrence of non-virologic failure, defined as any failure that does not meet the above virologic failure criteria (e.g. adverse event, lost to follow-up).
	To assess the safety of 12 weeks RDV-SOF in subjects with chronic HCV infection and no cirrhosis, and 24 weeks RDV-SOF in subjects with compensated cirrhosis.		**Safety endpoints**:<br>Occurrence of premature treatment discontinuation and occurrence of premature study discontinuation (overall and by reason for premature discontinuation).<br>Time to premature treatment discontinuation and time to premature study discontinuation*.<br>Occurrence of the following events (overall, by system organ class and by severity): TEAE considered to be at least possibly related to at least one of the study drugs, TEAE leading to premature treatment discontinuation, TE laboratory abnormality, grade 3/4 TEAE, TE SAE and death.<br>Time to first TEAE*, time to first grade 3/4 TEAE*<br>Time to first TE SAE*.
	To study the pharmacokinetics of SOF and RDV, evaluate potential drug-drug interactions with antiretrovirals and, as needed, interactions with concomitant prescribed or non-prescribed drugs.		Pharmacokinetic parameters of ravidasvir (and sofosbuvir if needed): Cmax, Tmax, pre-dose (C0), C24, Cmin, AUC0-24, and apparent oral clearance (CL/F) *.<br>Antiretroviral plasmatic concentration and Ratio week 4/ day 1 of tenofovir, emtricitabine, efavirenz, and nevirapine in HIV-HCV coinfected subjects treated with RDV-SOF.
	To describe subjects demographic, clinical and biological characteristics and their relationship with SVR12.		Baseline factors associated with SVR12 outcome.

	To assess subjects quality of life before and after therapy.		Change in the PROQOL-HCV domain scores from treatment initiation to 12 weeks after treatment completion*.
	To evaluate the presence of viral resistance-associated variants to RDV-SOF at the time of failure or at first point after failure when viral load is sufficient to get a positive result in patients with virological failure and their persistence until 1 year after treatment cessation or the initiation of an alternative HCV therapy.		Baseline HCV NS5A sequences and changes from treatment initiation in subjects not achieving SVR12.

RDV-SOF = ravidasvir plus sofosbuvir; HCV = hepatitis C virus; SVR12 = sustained virologic response 12 weeks after end of treatment; LLOQ = lower limit of quantification (defined as HCV RNA by PCR < 12 iu/ml or < 15 iu/ml); TEAE = Treatment Emergent Adverse Event; SAE = serious adverse event; PROQOL= Professional Quality of Life Scale. *This endpoint is not reported in the manuscript

STORM-C-1 intensive pharmacokinetic study of ravidasvir

Within the main trial, a formal intensive pharmacokinetic (PK) sub-study was designed to assess the steady-state PK of ravidasvir in HCV mono-infected subjects. The goal of this intensive PK sub-study was to generate robust PK data in Asian adults receiving 200 mg of ravidasvir once daily in combination with sofosbuvir 400 mg once daily. In addition, it was anticipated that the full concentration versus time profiles generated in the intensive PK study would aid the development of the population PK model planned at the end of the study.

Findings: A total of 28 HCV-monoinfected adults enrolled in the main trial provided additional consent for inclusion in the intensive PK study. All subjects were enrolled in Hospital Ampang in Malaysia. A total of 25 subjects had evaluable PK samples available for analysis: 21 were male (84%), 21 subjects were with cirrhosis and 4 subjects were without cirrhosis (all males). The median age (range) was 49.2 (21.2-64.0) years old, weight 65.5 (46.2-88.3) kg and body mass index 23.3 (18.3-30.9). The mean (SD) AUC_{0-24}, C_{max}, C_{last} of ravidasvir were 19.92 (12.77) hr.µg/mL, 2.54 (1.21) µg/mL and 0.19 (0.20) µg/mL, respectively. The median ravidasvir Tmax (range) was 1.8 (1.0-3.1) hours. Only 4 subjects with liver cirrhosis were included in the intensive PK study so a formal comparison with non-cirrhotic subjects was not possible but in an exploratory analysis no major differences in RDV concentrations plots were observed.

STORM-C-1 ravidasvir drug level measurements: sparse pharmacokinetic sampling

In Stage 1, all subjects (except those 25 who participated in the intensive PK substudy) had sparse pharmacokinetic blood sampling performed at Week 4, Week 8 and Week 12. At Week 4, two blood samples were drawn, one pre-dose and one 2-4 hours post-dose; at Week 8 a single blood sample was drawn at any time post-dose; and at Week 12, two blood samples were drawn 2 hours apart between 6 and 26 hours post-dose (a total of 5 blood samples per subject).

Findings: Overall, 296 adults (217 without and 79 with cirrhosis) had at least one plasma sample with ravidasvir concentration data available. A total of 271 subjects with 1,340 sparse PK blood samples (540 samples at Week 4, 269 samples at Week 8, and 531 samples at Week 12) were tested. Ravidasvir plasma concentrations in adults were comparable between the intensive and sparse PK sampling groups. At the study visits where ravidasvir plasma concentration were determined the values for subjects with virological failures were within the same observed range for those subjects who achieved SVR12. A formal population PK analysis to identify sources of inter-subject variability, such as individual subject characteristics and explore potential drug-drug interactions (e.g. antiretroviral co-treatment) will be conducted once Stage 2 sparse data are available.

Individual RDV plasma concentration versus time curves for 25 subjects in the intensive PK study for STORM-C-1

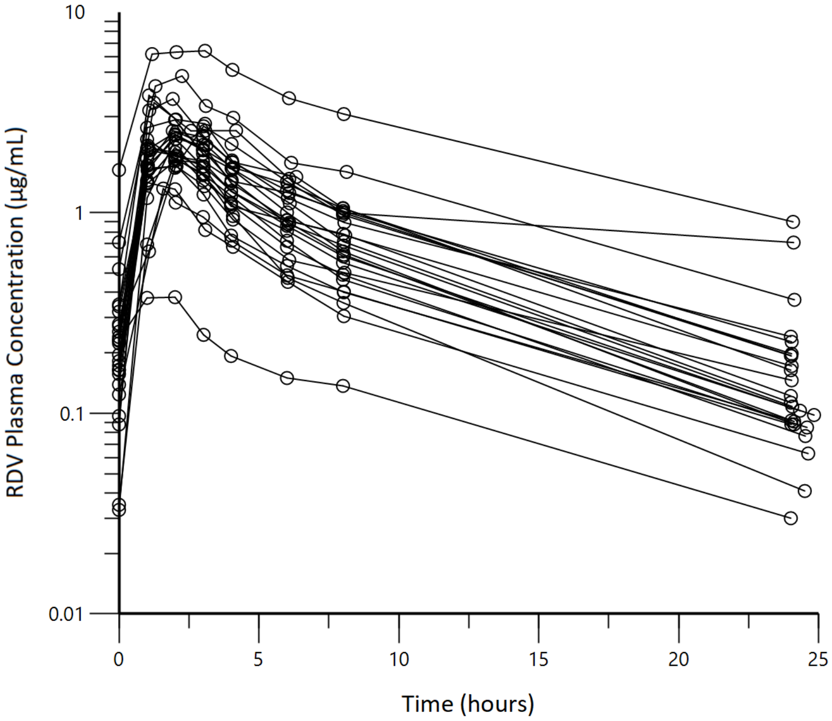

STORM-C-1: summary of ravidasvir pharmacokinetic parameters in all subjects (N=25)

	Tmax (hr)	Cmax (μg/mL)	Clast (μg/mL)	AUC_{0-24} (hr*μg/mL)	Cmin (μg/mL)	CLss_F (L/hr)
**N**	25	25	25	25	25	25
**Mean**	1.82	2.54	0.19	19.92	0.26	13.99
**SD**	0.57	1.21	0.20	12.77	0.32	11.24
**CV%**	31.4	47.6	106.9	64.1	122.1	80.4
**Min**	1.00	0.38	0.03	3.18	0.03	2.86
**Median**	2.00	2.33	0.11	17.32	0.17	11.55
**Max**	3.07	6.41	0.90	69.93	1.63	62.87

Subjects with major protocol deviations in the STORM-C-1 study

Group	Age (years)	Sex	Weight (Kg)	Description
NC	56	Male	46	QTc > 450ms
C	48	Male	80	QTc > 450ms
C	62	Female	61	Direct bilirubin > 3 x ULN
C	54	Male	87	Direct bilirubin > 3 x ULN<br>QTc > 450ms (TBC)
C	53	Male	74	Direct bilirubin > 3 x ULN
NC	60	Female	58	Corticosteroid use within 6 months before recruitment
NC	34	Male	64	Direct bilirubin > 3 x ULN

C: Cirrhotic. NC: Non-cirrhotic. QTc: Corrected QT interval. ULN: Upper limit of normality

Sustained virologic response 12 weeks after end of treatment by subgroup – full analysis set (N=300) in the STORM-C-1 study

**Variable**	**Category**	**SVR12**	**95% CI of SVR12**
Sex	Male	225/230 (98%)	95% to 99%
	Female	66/70 (94%)	86% to 98%
Baseline age (years)	<50	168/173 (97%)	93% to 99%
	≥50	123/127 (97%)	92% to 99%
Ethnicity	Malay	117/119 (98%)	94% to >99%
	Thai	76/79 (96%)	89% to 99%
	Other*	98/102 (96%)	90% to 99%
Region	Malaysia	214/219 (98%)	95% to 99%
	Thailand	77/81 (95%)	88% to 99%
Baseline body mass index (kg/m^2)	<25	185/191 (97%)	93% to 99%
	≥25	106/109 (97%)	92% to 99%
Cirrhosis	Yes	78/81 (96%)	90% to 99%
	No	213/219 (97%)	94% to 99%
HCV genotype	Genotype 1	123/124 (99%)	96% to 100%
	Genotype 2	2/2 (100%)	16% to 100%
	Genotype 3	153/158 (97%)	93% to 99%
	Genotype 6	13/16 (81%)	54% to 96%
HCV genotype and subtype	Genotype 1a	96/97 (99%)	94% to 100%
	Genotype 1b	27/27 (100%)	87% to 100%
	Genotype 2a	1/1 (100%)	3% to 100%
	Genotype 2b	1/1 (100%)	3% to 100%
	Genotype 3 (unknown subtype)	3/3 (100%)	29% to 100%
	Genotype 3a	133/138 (96%)	92% to 99%
	Genotype 3b	17/17 (100%)	80% to 100%
	Genotype 6a	1/1 (100%)	3% to 100%
	Genotype 6f	2/2 (100%)	16% to 100%
	Genotype 6i	1/1 (100%)	3% to 100%
	Genotype 6n	8/10 (80%)	44% to 97%
	Genotype 6t	0/1 (0%)	0% to 98%
	Genotype 6u	1/1 (100%)	3% to 100%
Baseline HCV RNA (IU/mL)	<800,000	80/85 (94%)	87% to 98%

Variable	Category	SVR12	95% CI of SVR12
	≥800,000	211/215 (98%)	95% to 99%
Baseline ALT	<1.5xULN	141/147 (96%)	91% to 98%
	≥1.5xULN	150/153 (98%)	94% to >99%
Baseline AST	<1.5xULN	146/151 (97%)	92% to 99%
	≥1.5xULN	145/149 (97%)	93% to 99%
IL28B	CC	213/221 (96%)	93% to 98%
	CT	77/78 (99%)	93% to 100%
	TT	1/1 (100%)	3% to 100%
Prior HCV treatment	Yes	95/99 (96%)	90% to 99%
	No	196/201 (98%)	94% to 99%
HIV co-infection	Yes	87/90 (97%)	91% to 99%
	No	204/210 (97%)	94% to 99%
Reported past injection drug use	Yes	129/133 (97%)	92% to 99%
	No	162/167 (97%)	93% to 99%
Reported attendance to an opioid substitution program or an oral substitution treatment (OST) †	Yes, currently	20/22 (91%)	71% to 99%
	Yes, in the past	19/19 (100%)	82% to 100%
	No	89/91 (98%)	92% to 100%
	Unknown	1/1 (100%)	3% to 100%
Adherence to study regimen	<90%	3/7 (43%)	10% to 82%
	≥90%	288/293 (98%)	96% to 99%
Completed study treatment	Yes	289/294 (98%)	96% to 99%
	No	2/6 (33%)	4% to 78%

*Other includes: Chinese, Lao, Burmese, Indian, Bumiputera Sabah, Bumiputera Sarawak, Portuguese, Lahu, and Sikh. †Question asked only to subjects who reported injection drug use. SVR12 = sustained virologic response 12 weeks after end of treatment.

Prevalence of specific baseline resistance associated variants in subjects in the STORM-C-1 study according to genotype and SVR12 rates

Genotype	NS5A RAV	RDV EC50 fold change [4].	SVR12 n/N (%)
1a	28V	1	4/4 (100%)
	28T	151	1/1 (100%)
	30H	108	1/1 (100%)
	31M	169	1/1 (100%)
	58D	140	1/1 (100%)
	93F	NA	1/1 (100%)
	93H	1295	1/1 (100%)
	93N	3068	1/1 (100%)
	30H+93H	2460	2/2 (100%)
	28A+58R	NA	1/1 (100%)
	28V+30H+93H	NA	1/1 (100%)
	28T+30L+93H	25120	1/1 (100%)
1b	93H	109	7/7 (100%)
	31M	20	1/1 (100%)
	30Q	1	1/1 (100%)
	92T+93H	15368	1/1 (100%)
	31M+93H	NA	1/1 (100%)
2a	31M	247	1/1 (100%)
2b	31M	NA	1/1 (100%)
3a	93H	546	16/18 (88.9%)

**Genotype**	**NS5A RAV**	**RDV EC50 fold change** [4].	**SVR12 n/N (%)**
	62L	NA	5/5 (100%)
	30S	NA	2/2 (100%)
	28K	NA	1/1 (100%)
	62L+93H	NA	5/5 (100%)
3b	30K+31M	NA	13/13 (100%)
	30K	NA	1/1 (100%)
	31M	NA	3/3 (100%)
6n	28V+93S	NA	8/10 (80%)
6t	28V+93S	NA	0/1 (0%)
6f	58S	NA	1/1 (100%)
6xa	28V	NA	1/1 (100%)
6i	28V	NA	1/1 (100%)

NA = not available

Treatment-emergent serious adverse events starting during treatment in subjects in the STORM-C-1 study

Group	System Organ Class / Preferred Term	Grade of severity	Day of onset after treatment initiation	Relation to study drugs
RDV-SOF 12 wks (non-cirrh.)	Infections and infestations/ Upper respiratory tract infection	1	6	Unlikely to be related
RDV-SOF 24 wks (cirrh.)	Hepatobiliary disorders/Bile duct stone	1	115	Not related
RDV-SOF 24 wks (cirrh.)	Musculoskeletal and connective tissue disorders/ Musculoskeletal chest pain	2	52	Not related
RDV-SOF 12 wks (non-cirrh.)	Infections and infestations/ Influenza	3	72	Unlikely to be related
RDV-SOF 24 wks (cirrh.)	Infections and infestations/Dengue fever	3	153	Not related
RDV-SOF 24 wks (cirrh.)	Infections and infestations/ Intervertebral discitis	3	163	Unlikely to be related
RDV-SOF 12 wks (non-cirrh.)	Infections and infestations/ Abdominal abscess & Renal and urinary disorders/ Urinary bladder rupture	3	13	Not related
RDV-SOF 12 wks (non-cirrh.)	Renal and urinary disorders/ Acute kidney injury	3	59	Possibly related to study drug*
RDV-SOF 24 wks (cirrh.)	Investigations/ Alanine aminotransferase increased	4	1	Not related

*Assessed by investigator as possibly due to a combination of Tenofovir-related renal tubular dysfunction and enalapril-induced hypotension and assessed as possibly related to RDV-SOF more as an indirect effect by increasing tenofovir concentration. Sponsor disagreed with assessment of causality with RDV/SOF. Post clinical DB lock, during CSR finalization, the investigator changed his assessment of causality for RDV to not related. TESAE therefore assessed by reporter to SOF & TDF, but not to RDV. RDV-SOF = ravidasvir plus sofosbuvir

Treatment-emergent serious adverse events starting after treatment in subjects in the STORM-C-1 study

Group	System Organ Class / Preferred Term	Grade of severity	Relation to study drugs
RDV-SOF 12 wks (non-cirrh.)	Eye disorders /Pterygium	2	Not related
RDV-SOF 12 wks (non-cirrh.)	Nervous system disorders/ Facial paralysis	3	Not related
RDV-SOF 12 wks (non-cirrh.)	Injury, poisoning and procedural complications/ Humerus fracture & Radial head dislocation	3	Not related
RDV-SOF 12 wks (non-cirrh.)	Injury, poisoning and procedural complications/ Intentional overdose	2	Not related
RDV-SOF 24 wks (cirrh.)	Cardiac disorders/ Acute myocardial infarction & Cardiogenic shock	3	Not related

RDV-SOF 24 wks (cirrh.)	Eye disorders/ Retinal detachment	2	Not related
RDV-SOF 24 wks (cirrh.)	Investigations/ Electrocardiogram ambulatory	1	Not related
RDV-SOF 24 wks (cirrh.)	Gastrointestinal disorders/ Oesophageal varices haemorrhage	3	Not related
RDV-SOF 24 wks (cirrh.)	Gastrointestinal disorders/ Pancreatitis acute	3	Not related
RDV-SOF 12 wks (non-cirrh.)	Infections and infestations/ Bronchitis	3	Not related
RDV-SOF 12 wks (non-cirrh.)	Injury, poisoning and procedural complications/ Radius fracture	3	Not related
RDV-SOF 12 wks (non-cirrh.)	Neoplasms benign, malignant and unspecified (incl cysts and polyps)/Adenocarcinoma of colon	3	Not related
RDV-SOF 12 wks (non-cirrh.)	Injury, poisoning and procedural complications/ Foot fracture	3	Not related

RDV-SOF = ravidasvir plus sofosbuvir

Antiretroviral pharmacokinetics in HIV Co infected Subjects (N=90) in the STORM-C-1 study

**Drug**	**ARV concentrations (ng/ml)**		**Ratio**
	**Without RDV-SOF (Day 1)**	**With RDV-SOF (Week 4)**	**Concentration Week 4/Day 1**
Tenofovir (N=47)	0·09 (0·02-0·25)	0·10 (0·05-0·28)	1·08 (0·54-3·70)
Emtricitabine (N=34)	0·38 (0·04-0·96)	0·39 (0·08-0·89)	1·00 (0·35-5·81)
Efavirenz (N=51)	2·58 (1·17-22·54)	2·54 (1·27-15·78)	0·96 (0·51-2·10)
Nevirapine (N=7)	9·52 (3·54-18·31)	7·68 (2·92-16·28)	0·91 (0·65-1·10)

ARV = antiretroviral; RDV-SOF = ravidasvir plus sofosbuvir; N = number of subjects; HIV = human immunodeficiency virus;

PROQOL-HCV Domain Scores and Change from Baseline by Visit - Safety Analysis Set in the STORM-C-1 study

		RDV+SOF 12 wks (non-cirrh.)				RDV+SOF 24 wks (cirrh.)				Overall			
**Domain**	**Visit**	**N**	**Mean (SD)**	**Median (Q1, Q3)**	**Min, Max**	**N**	**Mean (SD)**	**Median (Q1, Q3)**	**Min, Max**	**N**	**Mean (SD)**	**Median (Q1, Q3)**	**Min, Max**
Physical health	Baseline	219	68.9 (15.3)	71 (63, 83)	8, 88	80	62.6 (16.1)	63 (50, 75)	21, 88	299	67.2 (15.7)	71 (58, 79)	8, 88
	Follow-up Week 12	218	72.4 (13.0)	75 (63, 83)	29, 92	80	64.8 (15.8)	67 (54, 79)	17, 88	298	70.4 (14.2)	75 (58, 83)	17, 92
	Change at Follow-up Week 12	217	3.5 (12.6)	4 (-4, 13)	-33, 33	79	2.3 (13.4)	0 (-4, 8)	-29, 38	296	3.2 (12.8)	0 (-4, 10)	-33, 38
Emotional health	Baseline	220	76.5 (19.4)	81 (64, 92)	11, 100	81	72.0 (21.6)	72 (64, 89)	6, 100	301	75.3 (20.1)	81 (64, 92)	6, 100
	Follow-up Week 12	218	82.5 (17.2)	88 (75, 97)	19, 100	80	77.2 (20.7)	82 (64, 94)	0, 100	298	81.1 (18.3)	86 (72, 97)	0, 100
	Change at Follow-up Week 12	218	6.0 (16.0)	6 (-3, 14)	-44, 53	80	5.3 (18.3)	3 (-3, 13)	-39, 78	298	5.8 (16.6)	6 (-3, 14)	-44, 78
Future uncertainty	Baseline	220	70.5 (21.4)	71 (58, 88)	4, 100	81	68.2 (23.2)	67 (50, 88)	13, 100	301	69.8 (21.9)	71 (54, 88)	4, 100
	Follow-up Week 12	218	78.6 (20.5)	83 (67, 96)	0, 100	80	77.3 (20.0)	79 (67, 96)	13, 100	298	78.2 (20.3)	83 (67, 96)	0, 100
	Change at Follow-up Week 12	218	8.2 (19.5)	6 (-4, 21)	-46, 63	80	9.4 (20.5)	8 (-2, 23)	-50, 58	298	8.5 (19.7)	8 (-4, 21)	-50, 63
Intimate relationships	Baseline	219	82.2 (21.0)	92 (67, 100)	0, 100	80	75.6 (24.2)	79 (62, 100)	0, 100	299	80.4 (22.0)	83 (67, 100)	0, 100
	Follow-up Week 12	218	86.7 (19.4)	100 (75, 100)	8, 100	79	79.4 (21.6)	83 (67, 100)	0, 100	297	84.8 (20.2)	92 (75, 100)	0, 100

Abbreviations: Max = maximum; Min = minimum;N = number of subjects; RDV SOF = ravidasvir plus sofosbuvir; SD = standard deviation; Q = quartile.

Time to First Treatment-Emergent Adverse Event of ≥Grade 3 – SafetyAnalysis Set in the STORM-C-1 study

Parameter	Category	RDV-SOF 12 wks (non-cirrh.)	RDV-SOF 24 wks (cirrh.)
Time to first TEAE of ≥Grade 3 (weeks)	N	13	8
	Mean (SD)	12.9 (12.6)	22.3 (20.2)
	Median (Q1, Q3)	8.4 (4.1, 19.4)	24.8 (0.1, 39.4)
	Min, Max	0.1, 35.0	0.1, 49.9
Kaplan-Meier cumulative probability(95% CI) of experiencing a firstTEAE of ≥Grade 3:	1 week after HCVtreatment initiation	0.9% (0.2% to 3.6%)	3.7% (1.2% to 11.0%)
	4 weeks after HCV treatment initiation	1.4% (0.4% to 4.2%)	3.7% (1.2% to 11.0%)
	8 weeks after HCV treatment initiation	2.7% (1.2% to 6.0%)	3.7% (1.2% to 11.0%)
	12 weeks after HCV treatment initiation	3.7% (1.8% to 7.2%)	3.7% (1.2% to 11.0%)
	16 weeks after HCV treatment initiation	3.7% (1.8% to 7.2%)	3.7% (1.2% to 11.0%)
	24 weeks after HCV treatment initiation	4.6% (2.5% to 8.3%)	4.9% (1.9% to 12.6%)
	36 weeks after HCV treatment initiation	6.0% (3.5% to 10.0%)	6.2% (2.6% to 14.2%)
	48 weeks after HCV treatment initiation	-	8.7% (4.2% to 17.3%)

Abbreviations: CI = confidence interval; HCV = hepatitis C virus; Max = maximum; Min = minimum;N = number of subjects; RDV SOF = ravidasvir plus sofosbuvir; SD = standard deviation; TEAE = treatment-emergent adverse event; Q = quartile; wks = weeks.

Time to premature treatment discontinuation – Safety Analysis Set in the STORM-C-1 study

**Parameter**	**Category**	**RDV-SOF 12 wks (non-cirrh.)**	**RDV-SOF 24 wks (cirrh.)**
Time to premature treatment discontinuation (weeks)	N	3	3
	Mean (SD)	2.9 (4.4)	7.4 (11.4)
	Median (Q1, Q3)	0.6 (0.1, 8.0)	1.3 (0.3, 20.6)
	Min, Max	0.1, 8.0	0.3, 20.6
Kaplan-Meier cumulative probability(95% CI) of prematurely discontinuing treatment	1 week after HCVtreatment initiation	0.9% (0.2% to 3.6%)	1.2% (0.2% to 8.4%)
	4 weeks after HCV treatment initiation	0.9% (0.2% to 3.6%)	2.5% (0.6% to 9.5%)
	8 weeks after HCV treatment initiation	1.4% (0.4% to 4.2%)	2.5% (0.6% to 9.5%)
	12 weeks after HCV treatment initiation	1.4% (0.4% to 4.2%)	2.5% (0.6% to 9.5%)
	16 weeks after HCV treatment initiation	-	2.5% (0.6% to 9.5%)
	20 weeks after HCV treatment initiation	-	2.5% (0.6% to 9.5%)
	24 weeks after HCV treatment initiation	-	3.7% (1.2% to 11.0%)

Abbreviations: CI = confidence interval; HCV = hepatitis C virus; Max = maximum; Min = minimum;N = number of subjects; RDV = ravidasvir; SD = standard deviation; SOF = sofosbuvir; Q = quartile;wks = weeks.

Time to First Treatment-Emergent Adverse Event Leading to Permanent Treatment Discontinuation – Safety Analysis Set in the STORM-C-1 study

**Parameter**	**Category**	**RDV-SOF 12 wks (non-cirrh.)**	**RDV-SOF 24 wks (cirrh.)**
Time to first TEAE leading topermanent treatment discontinuation (weeks)	N	1	2
	Mean (SD)	0.3 (-)	0.2 (0.1)
	Median (Q1, Q3)	0.3 (0.3, 0.3)	0.2 (0.1, 0.3)
	Min, Max	0.3, 0.3	0.1, 0.3
Kaplan-Meier cumulative probability (95% CI) of experiencing a first TEAE leading to permanent treatment discontinuation:	1 week after HCV treatment initiation	0.5% (0.1% to 3.2%)	2.5% (0.6% to 9.5%)
	4 weeks after HCV treatment initiation	0.5% (0.1% to 3.2%)	2.5% (0.6% to 9.5%)
	8 weeks after HCV treatment initiation	0.5% (0.1% to 3.2%)	2.5% (0.6% to 9.5%)
	12 weeks after HCV treatment initiation	0.5% (0.1% to 3.2%)	2.5% (0.6% to 9.5%)
	24 weeks after HCV treatment initiation	0.5% (0.1% to 3.2%)	2.5% (0.6% to 9.5%)
	36 weeks after HCV treatment initiation	0.5% (0.1% to 3.2%)	2.5% (0.6% to 9.5%)
	48 weeks after HCV treatment initiation	-	2.5% (0.6% to 9.5%)

Abbreviations: CI = confidence interval; HCV = hepatitis C virus; Max = maximum; Min = minimum;N = number of subjects; RDV = ravidasvir; SD = standard deviation; SOF = sofosbuvir; TEAE = treatment-emergent adverse event; Q = quartile; wks = weeks.

Time to Premature Study Discontinuation - Safety Analysis Set in the STORM-C-1 study

**Parameter**	**Category**	**RDV-SOF 12 wks (non-cirrh.)**	**RDV-SOF 24 wks (cirrh.)**
Time to premature study discontinuation (weeks)	N	2	1
	Mean (SD)	2.3 (2.4)	1.6 (-)
	Median (Q1, Q3)	2.3 (0.6, 4.0)	1.6 (1.6, 1.6)
	Min, Max	0.6, 4.0	1.6, 1.6
Kaplan-Meier cumulative probability (95% CI) of prematurely discontinuing the study:	1 week after HCV treatment initiation	0.5% (0.1% to 3.2%)	0.0% (-)
	4 weeks after HCV treatment initiation	0.9% (0.2% to 3.6%)	1.2% (0.2% to 8.4%)
	8 weeks after HCV treatment initiation	0.9% (0.2% to 3.6%)	1.2% (0.2% to 8.4%)
	12 weeks after HCV treatment initiation	0.9% (0.2% to 3.6%)	1.2% (0.2% to 8.4%)
	16 weeks after HCV treatment initiation	0.9% (0.2% to 3.6%)	1.2% (0.2% to 8.4%)
	20 weeks after HCV treatment initiation	0.9% (0.2% to 3.6%)	1.2% (0.2% to 8.4%)
	24 weeks after HCV treatment initiation	0.9% (0.2% to 3.6%)	1.2% (0.2% to 8.4%)
	28 weeks after HCV treatment initiation	-	1.2% (0.2% to 8.4%)
	32 weeks after HCV treatment initiation	-	1.2% (0.2% to 8.4%)
	36 weeks after HCV treatment initiation	-	1.2% (0.2% to 8.4%)
	40 weeks after HCV treatment initiation	-	1.2% (0.2% to 8.4%)
	44 weeks after HCV treatment initiation	-	1.2% (0.2% to 8.4%)
	48 weeks after HCV treatment initiation	-	1.2% (0.2% to 8.4%)

Abbreviations: CI = confidence interval; HCV = hepatitis C virus; Max = maximum; Min = minimum; N = number of subjects; RDV = ravidasvir; SD = standard deviation; SOF = sofosbuvir; Q = quartile; wks =weeks.

Time to First Treatment-Emergent SAE- Safety Analysis Set in the STORM-C-1 study

Parameter	Category	RDV-SOF 12 wks (non-cirrh.)	RDV-SOF 24 wks (cirrh.)
Time to first treatment-emergent serious adverse event (weeks)	N	11	9
	Mean (SD)	19.0 (12.2)	24.6 (19.6)
	Median (Q1, Q3)	21.6 (8.4, 28.9)	21.9 (7.4, 41.4)
	Min, Max	0.9, 35.6	0.1, 49.9
Kaplan-Meier cumulative probability(95% CI) of experiencing a first treatment-emergent serious adverse event	1 week after HCV treatment initiation	0.5% (0.1% to 3.2%)	2.5% (0.6% to 9.5%)
	4 weeks after HCV treatment initiation	0.9% (0.2% to 3.6%)	2.5% (0.6% to 9.5%)
	8 weeks after HCV treatment initiation	0.9% (0.2% to 3.6%)	3.7% (1.2% to 11.1%)
	12 weeks after HCV treatment initiation	1.8% (0.7% to 4.8%)	3.7% (1.2% to 11.1%)
	16 weeks after HCV treatment initiation	1.8% (0.7% to 4.8%)	3.7% (1.2% to 11.1%)
	20 weeks after HCV treatment initiation	2.3% (1.0% to 5.4%)	5.0% (1.9% to 12.7%)
	24 weeks after HCV treatment initiation	3.7% (1.9% to 7.2%)	6.2% (2.6% to 14.3%)
	28 weeks after HCV treatment initiation	3.7% (1.9% to 7.2%)	6.2% (2.6% to 14.3%)
	32 weeks after HCV treatment initiation	4.1% (2.2% to 7.8%)	6.2% (2.6% to 14.3%)
	36 weeks after HCV treatment initiation	5.0% (2.8% to 8.9%)	6.2% (2.6% to 14.3%)
	40 weeks after HCV treatment initiation	-	7.5% (3.4% to 15.9%)
	44 weeks after HCV treatment initiation	-	8.7% (4.3% to 17.4%)
	48 weeks after HCV treatment initiation	-	10.0% (5.1% to 19.0%)

Abbreviations: CI = confidence interval; HCV = hepatitis C virus; Max = maximum; Min = minimum;N = number of subjects; RDV = Ravidasvir; SD = standard deviation; SAE = serious adverse events; SOF = Sofosbuvir; Q = quartile; wks = weeks.
